# TNFα-mediated *Hsd11b1* binding of NF-κB p65 is associated with suppression of 11β-HSD1 in muscle

**DOI:** 10.1530/JOE-13-0494

**Published:** 2014-03

**Authors:** Craig L Doig, Jamila Bashir, Agnieszka E Zielinska, Mark S Cooper, Paul M Stewart, Gareth G Lavery

**Affiliations:** School of Clinical and Experimental MedicineCentre for Endocrinology, Diabetes and Metabolism, University of BirminghamBirmingham, B15 2TTUK

**Keywords:** glucocorticoid, inflammation, metabolism, muscle

## Abstract

The activity of the enzyme 11β-hydroxysteroid dehydrogenase type 1 (11β-HSD1), which converts inactive cortisone (11-dehydrocorticosterone (11-DHC)) (in mice) into the active glucocorticoid (GC) cortisol (corticosterone in mice), can amplify tissue GC exposure. Elevated TNFα is a common feature in a range of inflammatory disorders and is detrimental to muscle function in diseases such as rheumatoid arthritis and chronic obstructive pulmonary disease. We have previously demonstrated that 11β-HSD1 activity is increased in the mesenchymal stromal cells (MSCs) by TNFα treatment and suggested that this is an autoregulatory anti-inflammatory mechanism. This upregulation was mediated by the P2 promoter of the *Hsd11b1* gene and was dependent on the NF-κB signalling pathway. In this study, we show that in contrast to MSCs, in differentiated C2C12 and primary murine myotubes, TNFα suppresses *Hsd11b1* mRNA expression and activity through the utilization of the alternative P1 promoter. As with MSCs, in response to TNFα treatment, NF-κB p65 was translocated to the nucleus. However, ChIP analysis demonstrated that the direct binding was seen at position −218 to −245 bp of the *Hsd11b1* gene's P1 promoter but not at the P2 promoter. These studies demonstrate the existence of differential regulation of 11β-HSD1 expression in muscle cells through TNFα/p65 signalling and the P1 promoter, further enhancing our understanding of the role of 11β-HSD1 in the context of inflammatory disease.

## Introduction

The endogenous glucocorticoid (GC) concentrations are determined by the activity of the hypothalamic–pituitary–adrenal axis, with tissue and intracellular exposure further augmented through the activity of the enzyme 11β-hydroxysteroid dehydrogenase type 1 (11β-HSD1), which converts inactive cortisone (11-dehydrocorticosterone (11-DHC) in mice) to the active GC cortisol (corticosterone in mice) (Stewart 2003, 2005, Zhang *et al*. 2013). The ability of 11β-HSD1 to elevate cellular GC levels has led it to be implicated in the modulation of a number of metabolic and inflammatory disease processes (Hardy *et al*. 2008, Morgan *et al*. 2009, Kaur *et al*. 2010). Therefore, the identification of factors and mechanisms regulating 11β-HSD1 expression and activity could further highlight its physiological and pathophysiological roles.

Elevation of the pro-inflammatory cytokine TNFα is a cardinal feature of a range of inflammatory disorders (Bamias *et al*. 2013, Golikova *et al*. 2013, Khosravi *et al*. 2013, Moelants *et al*. 2013). We have previously reported that TNFα increases 11β-HSD1 expression and activity in the cells of the mesenchymal lineage, including osteoblasts and fibroblasts. In these cells, it was demonstrated that this was mediated through the classical *Hsd11b1* P2 promoter and that the induction of 11β-HSD1 activity by TNFα was dependent upon NF-κB signalling. However, no direct binding site of the NF-κB p65 subunit to the P2 promoter could be identified. Basal NF-κB signalling plays a fundamental role in skeletal muscle myogenesis; however, during prolonged inflammatory stress, TNFα-mediated NF-κB activity can abrogate myogenesis, inhibiting differentiation and increasing catabolic processes contributing to muscle wasting (Yamaki *et al*. 2012). Previous research has demonstrated down-regulation of 11β-HSD1 activity when mature C2C12 muscle cell myotubes are stimulated with TNFα and it was proposed that NF-κB binding to sites in the *Hsd11b1* promoter may be directly responsible for this regulation (Aubry & Odermatt 2009).

In this study, we demonstrate that in contrast to TNFα upregulation of 11β-HSD1 in mesenchymal stromal cells (MSCs) acting through indirect NF-κB regulation at the P2 promoter, in C2C12 and primary murine myotubes TNFα stimulates the NF-κB p65 subunit to bind the alternate *Hsd11b1* P1 promoter and mediate inhibition of 11β-HSD1 activity.

## Materials and methods

### C2C12 cell culture

Mouse skeletal muscle cell line C2C12 (European Collection of Cell Cultures (ECACC), Salisbury, Wiltshire, UK ) myoblasts were maintained in DMEM (PAA Laboratories, Yeovil, Somerset, UK), high-glucose, supplemented with FBS (10%) penicillin/streptomycin (ten units) and incubated at 37 °C in the presence of 5% CO_2_. The media were replaced for every 48 h and the cells were split three times weekly. To differentiate myoblasts into myotubes, they were cultivated to 70% confluence before addition of DMEM, high-glucose, supplemented with 5% horse serum and penicillin/streptomycin (ten units), and the media were replaced every 48 h.

### Primary mouse muscle cell culture

Primary muscle dissection and culture of myotubes derived from muscle satellite cells were conducted as reported by Rosenblatt *et al*. (1995). Briefly, extensor digitorum longus (EDL) was dissected from mice at 5 weeks of age. These were digested in type 1 collagenase for 2 h. Individual fibres of the muscle were then disrupted gently using a glass pipette. Individual muscle fibre was then placed into 24-well plates coated with Matrigel and left for 72 h. After 72 h the satellite cells had migrated from the fibre and the fibres were removed from the plate, satellite cell proliferation media were added. Satellite cells-derived myoblasts were then cultured to confluence and differentiated to myotubes stable in culture for ∼14 days. Treatments were for 24 h unless otherwise indicated and occurred in serum-free media using murine TNFα (10 ng/ml); the vehicle control used was 0.1% BSA and dexamethasone (1 μM).

### RNA extraction, RT-PCR and quantitative real-time PCR

Total RNA was isolated and extracted from cell lysates using TRI-Reagent (Sigma–Aldrich). The quality and quantity of RNA recovered were assessed by running on 1.5% agarose gel and Nanodrop spectrophotometer. RT-PCR used 1 μg RNA per sample and was carried out using the reverse transcription kit (CODE) Applied Biosciences (kit code is 4368814). Specific mRNA levels were determined using an ABI 7900 sequence detection system (Applied Biosystems). Reactions were carried out in 12.5 μl volumes on 384 well plates (Applied Biosystems) in a reaction buffer containing 2× Taqman Universal PCR Master Mix (Applied Biosystems). Primers and probes for specific genes were purchased in ‘Assay on Demand’ format from Applied Biosystems (IGF1:Mm00439560_m1, MYOD:Mm00521984_m1,TP53:Mm00480750_m1, H6PD:Mm00557617). These were normalised against 18S rRNA (Applied Biosystems) as an internal control. Raw data were recovered as *C*T values and analysed as per the 2^−ΔΔ*C*T^ method.

RT-PCR of alternative transcripts from the *Hsd11b1* gene was carried out as described in Staab *et al*. (2011).

### Western immunoblotting

Protein lysates were collected in RIPA buffer (50 mmol/l Tris pH 7.4, 1% NP-40, 0.25% sodium deoxycholate, 150 mmol/l NaCl, 1 mmol/l EDTA), 1 mmol/l phenylmethylsulfonyl fluoride and protease inhibitor cocktail (Roche), stored at −80 °C (30 min), defrosted on ice and centrifuged at 4 °C (10 min, 11 269 ***g***). The supernatant was recovered and total protein concentration was assessed by Bio-Rad assay. Total proteins (25 μg) were resolved on a 12% SDS–PAGE gel and transferred onto a nitrocellulose membrane. Primary antibodies used were mouse anti-p65 (Santa Cruz Sc-8008), mouse anti-β-actin (Sigma–Aldrich A-5441), mouse anti-α-tubulin (Santa Cruz Sc-5286) and rabbit anti-11β-HSD1 (Ricketts *et al*. 1998).

Secondary antibodies (Dako, Ely, Cambridgeshire, UK) anti-mouse and anti-rabbit conjugated with HRP were added at a dilution of 1/5000. Equal loading of protein content was verified using β-actin and the bands were visualised using ECL detection system (GE Healthcare, Little Chalfont, Buckinghamshire, UK).

### Chromatin immunoprecipitation

Chromatin immunoprecipitation was conducted using the EZ-ChIP kit from Millipore (Watford, Hertfordshire, UK). The C2C12 myotubes were differentiated in T175 volumes flasks. The cells were cross-linked with formaldehyde (1%) for 10 min at room temperature. Glycine at a final concentration of 125 mM was added to quench and left at room temperature for 5 min. The flasks were washed twice in ice-cold PBS and sonicated using a Bioruptor (Diagenode, Seraing, Belgium) to produce chromatin smears with an average size of 500–1000 bp. Chromatin immunoprecipitations were carried out using the anti-p65 (Santa Cruz Sc-8008) alongside IgG control. DNA recovery was conducted and SYBR green PCR was carried out using the primer sequences described in Table 1.

### 11β-HSD1 activity assay

Briefly, the cells were incubated with 100 nmol/l 11-DHC and tritiated tracer (^3^HA) made in-house was added to each well at 0.22 μCi/reaction. Steroids were then extracted using dichloromethane, separated using a mobile phase consisting of ethanol and chloroform (8:92) by thin layer chromatography and scanned using a Bioscan 3000 image analyser (Lablogic, Sheffield, South Yorkshire, UK). The calculations of 11-DHC (A) to corticosterone (B) were conducted as follows B/(A+B)×100=% Conversion.

### Statistical analysis

Data shown are mean±s.e.m. of at least three independent experiments with statistical significance defined as *P*<0.05 (*P<0.05; **P<0.01; ***P<0.001) using unpaired Student's *t*-test and were conducted with Prism (GraphPad, La Jolla, CA, USA). Statistical analysis on real-time PCR data was carried out on mean Δ*C*t values.

## Results

### TNFα induces p65 nuclear translocation in C2C12 cells

**Figure 1:**
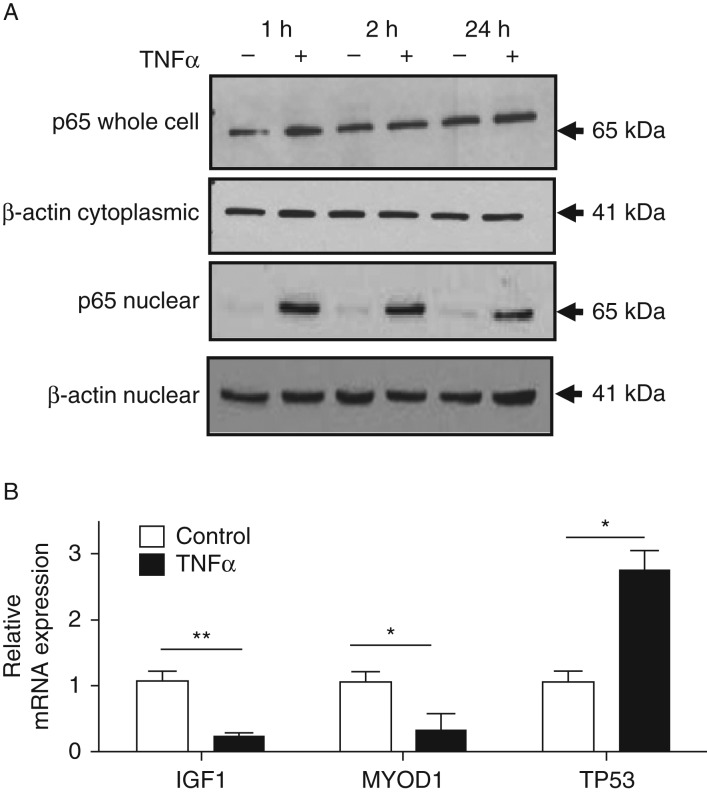
Archetypal response of C2C12 myotubes to TNFα. (A) Western blot analysis of p65 expression in C2C12 myotubes for 1, 2 and 24 h post treatment with either 0.1% BSA as control or TNFα (10 ng/ml). (B) Real-time PCR analysis of C2C12 myotube response to TNFα treatment (*P<0.05, **P<0.01).

Treatment of cells with TNFα (10 ng/ml) for 1 or 2 h produced no overall change in p65 cell content. However, isolation of the nuclear fraction showed enrichment of p65 in the nucleus upon TNFα stimulation, validating a functional NF-κB signalling pathway in 5-day-differentiated C2C12 myotubes (Fig. 1A). To assess archetypal responses to TNFα, mRNA levels were examined for the cell-cycle arrest gene *Tp53* (*Trp53*) and pro-growth pro-differentiation genes *Igf1* and *Myod1* (Fig. 1B). TNFα treatment induced a significant increase in TP53 and significant decrease in *Igf1* and *Myod1* levels, validating the functionality of the model as has been described previously (Frost *et al*. 2003, Langen *et al*. 2004, Schwarzkopf *et al*. 2006).

### TNFα suppresses 11β-HSD1 mRNA and protein expression

**Figure 2:**
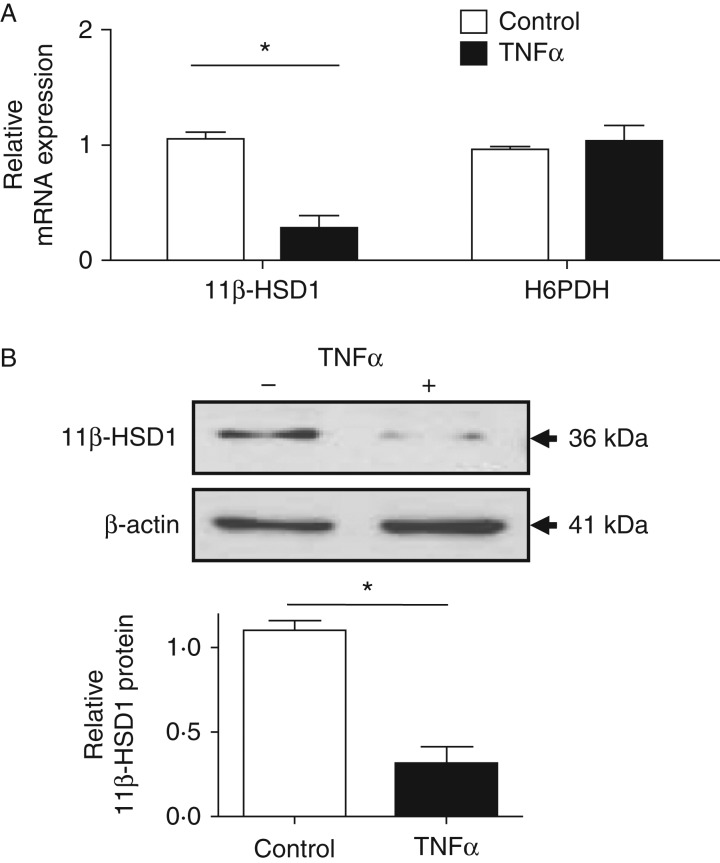
TNFα suppresses 11β-HSD1 mRNA and protein expression. (A) Real-time PCR analysis of 11β-HSD1 and H6PDH mRNA in C2C12 myotubes treated for 24 h with TNFα. (B) Western immunoblot analysis of p65 and β-actin from C2C12 myotubes treated for 24 h with TNFα compared to control. Densitometry of the western immunoblots were carried out using ImageJ (*P<0.05).

TNFα treatment of C2C12 myotubes decreases 11β-HSD1 mRNA in comparison with controls. Furthermore, analysis of *H6pdh* (*H6pd*) mRNA which is the enzyme that provides NADPH to support 11β-HSD1 activity showed no change (Fig. 2A; Lavery *et al*. 2006). To establish if this response was detectable at a protein level, myotubes were challenged with TNFα for 24 h and were examined for levels of 11β-HSD1 protein. TNFα treatment produced a significant decrease in the expression of 11β-HSD1 protein within myotubes, compared with vehicle control (Fig. 2B).

### TNFα suppresses 11β-HSD1 enzyme activity

**Figure 3:**
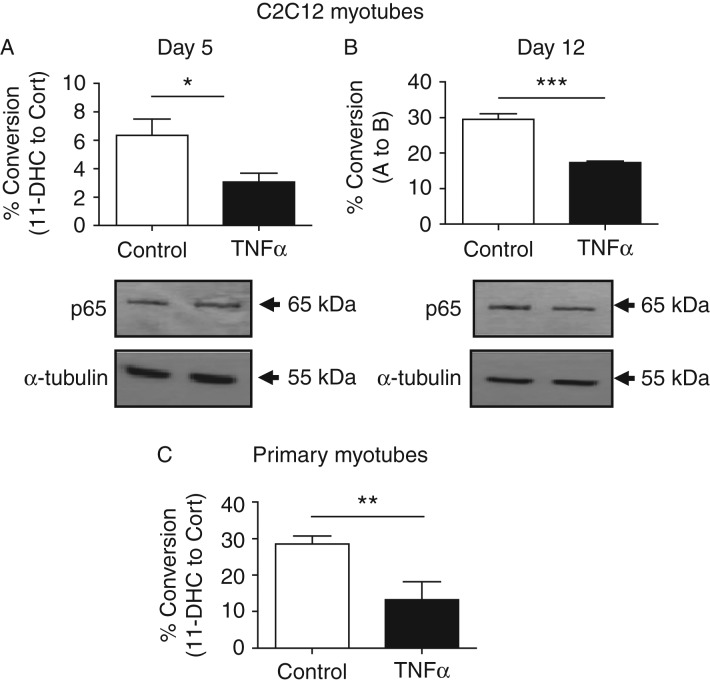
TNFα suppresses 11β-HSD1 activity in C2C12 and primary mouse myotubes. (A) C2C12 control cells were differentiated for 5 days, TNFα treatments were added 24 h before being assayed for 11β-HSD1 activity. (B) C2C12 control cells were differentiated for 12 days, TNFα treatments were added 24 h before being assayed for 11β-HSD1 activity. Protein lysates from C2C12 cells differentiated for 5 and 12 days (+/− TNFα for 24 h) were subject to western immunoblots demonstrating p65 expression levels. (C) Primary myotubes differentiated from mouse EDL muscle satellite cells were treated with TNFα or control for 24 h before conducting being assayed for 11β-HSD1 activity (*P<0.05, **P<0.01, ***P<0.001).

It is well established that 11β-HSD1 activity increases during the process of C2C12 differentiation into myotubes (Aubry & Odermatt 2009). In order to establish the relative functional impact of TNFα on 11β-HSD1 enzyme activity, C2C12 cells were differentiated for 4 days and activity assessed or vehicle/TNFα-treated for a further day (5 day total). The conversion of 11-DHC to corticosterone by 11β-HSD1 increased from day 4 to day 5 (Fig. 3A). However, TNFα treatment at day 4 of differentiation for an additional day significantly reduced 11β-HSD1 activity, suppressing it to the level of 4-day differentiated cells (Fig. 3A). We also tested cells with a greater degree of differentiation, examining C2C12 cells at day 11 and day 12 (Fig. 3B). The conversion rate increased significantly from day 11 to day 12 and TNFα treatment on day 12 suppressed 11β-HSD1 activity back towards that of day 11, confirming a robust effect equivalent to the effect seen at the earlier day 4 and 5 time points. To endorse these findings, we prepared 7-day differentiated myotubes from primary EDL muscle. Again, we saw a significant suppression of 11β-HSD1 activity following 1-day treatment with TNFα, consistent with the data from the fully differentiated C2C12 cells (Fig. 3C).

These data suggest that the suppression of 11β-HSD1 activity in response to TNFα is unlikely to be due solely to delays in differentiation, as C2C12 and primary muscle cells were fully differentiated and may indicate a direct effect of a TNFα-dependent factor acting at the *Hsd11b1* gene promoter to regulate the suppression of 11β-HSD1 expression.

### TNFα suppresses 11β-HSD1 at the level of transcription

**Figure 4:**
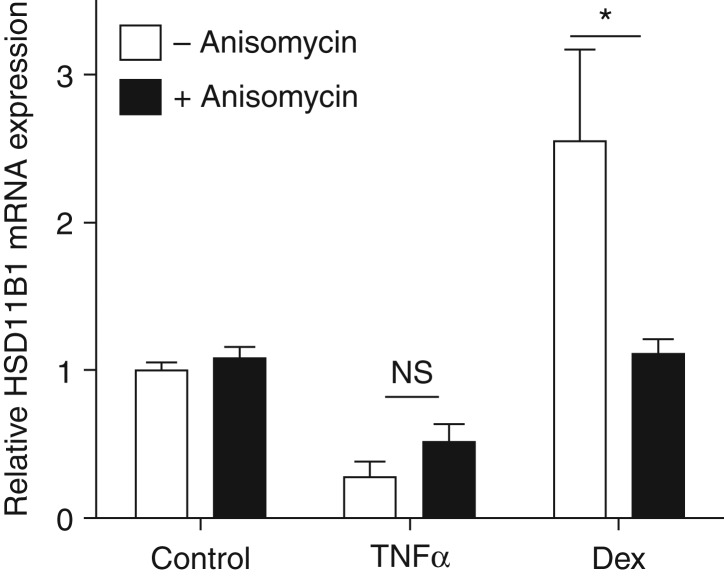
TNFα reduces 11β-HSD1 mRNA independent of the presence of anisomycin. Myotubes were treated with TNFα or dexamethasone in the presence and absence of the protein synthesis inhibitor anisomycin. 11β-HSD1 mRNA was measured by RT-PCR using ribosomal 18S as a standard (*P<0.05).

To establish if the downregulation of 11β-HSD1 occurred through a transcriptional mechanism, C2C12 myotubes were treated with TNFα in the presence and absence of anisomycin to inhibit protein synthesis. Accordingly, the suppression of 11β-HSD1 mRNA transcription in response to TNFα is unchanged in the presence of anisomycin, suggesting that the effect is primarily at the level of transcription and not the consequence of secondary protein synthesis (Fig. 4). We used dexamethasone as a control, as it is well known to induce 11β-HSD1 mRNA (Fig. 4). However, in the presence of anisomycin, the Dex-induced 11β-HSD1 increase is attenuated, indicating a requirement of secondary protein synthesis to elicit the response.

These data demonstrate that TNFα initiates activation of a factor not requiring protein synthesis that can act directly at the 11β-HSD1 promoter, and we hypothesised that this would most likely be the TNFα target NF-κB.

### The *Hsd11b1* P1 promoter contains a P65-binding site

**Figure 5:**
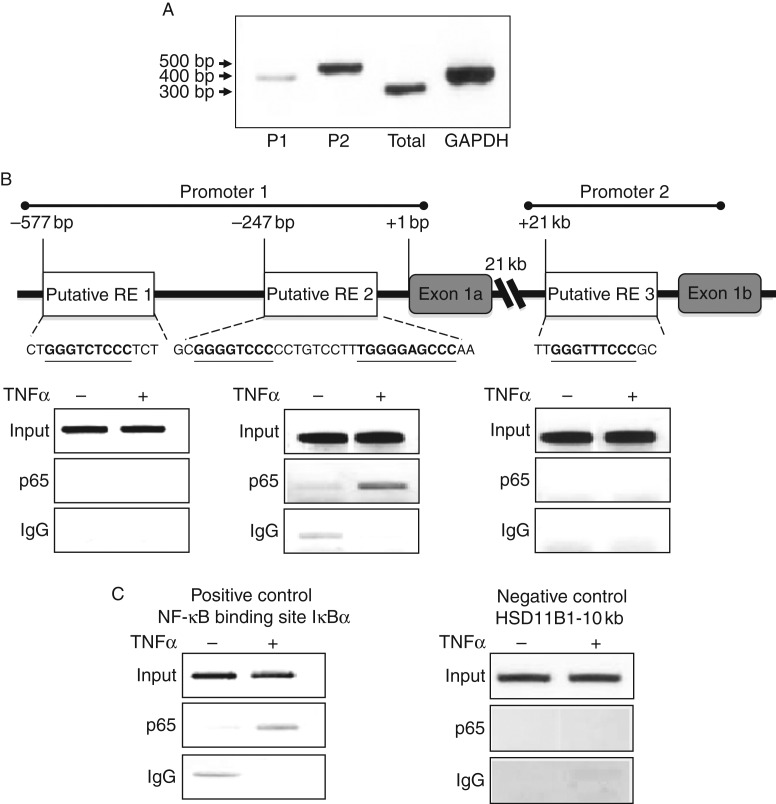
The NF-κB transcription factor p65 is recruited to the *Hsd11b1* P1 promoter upon stimulation by TNFα. (A) RNA isolated from C2C12 myotubes was assessed for the expression of *Hsd11b1* promoter 1 (P1), promoter 2 (P2), total and GAPDH transcripts by semi-quantitative RT-PCR. (B) Putative binding of p65 was measured using ChIP assays carried out on C2C12 myotubes. Fixed chromatin lysates were immuno-precipitated with p65 antibody and recovered DNA subject to PCR with primers containing putative p65-binding sites. Total chromatin served as input and IgG was used as a negative control, images are representative of biological triplicate experiments. (C) As a positive control a region for binding of p65 was assessed using an established NF-κB-binding site for IκBα. As an additional negative-control site a region 10 kb upstream was chosen to confirm the absence of specific binding.

Transcription of the *Hsd11b1* gene is regulated by two promoter regions: P1 and P2 (Bruley *et al*. 2006), and we confirmed that both promoters were active in our differentiated C2C12 cells, with promoter P2 usage preferential to promoter P1 (Fig. 5A). As we hypothesised that NF-κB binding may be associated with 11β-HSD1 suppression, we conducted ChIP analysis on C2C12 cells treated with TNFα, assessing p65 binding at putative response elements in the *Hsd11b1* P1 and P2 promoters. *In silico* analysis revealed three candidate sequences with resemblance to a consensus NF-κB-binding site (GGGACTTTCC), two of which occur within close proximity to each other and are located in the P1 promoter, upstream of exon 1a transcription start site, with all three depicted in Fig. 5B. ChIP analysis of samples amplified with primers for the putative p65-binding regions following p65 pull down indicated no enrichment of p65 binding in putative regions 1 and 3 in comparison with control IgG. However, putative region 2 demonstrates that TNFα induces an increase in the p65-binding levels in comparison with control and suggests active recruitment of the p65 protein to the *Hsd11b1* P1 promoter. As a positive control, TNFα-treated C2C12 cells were analysed by ChIP for enrichment of p65 to a well-validated IkBα-binding site, and confirmatory of NF-κB activation, further endorsing the finding of TNFα-mediated NF-κB-binding associated with suppression of 11β-HSD1 expression and activity (Fig. 5B). Additionally, a 10 kb upstream sequence of *Hsd11b1* was used to validate the absence of p65 as a negative control region and no TNFα enrichment was observed for this sequence (Fig. 5B).

## Discussion

Stimulation of 11β-HSD1 activity following TNFα exposure has been described for early progenitor cells of the mesenchymal lineage, particularly in cells of human origin (Zhang *et al*. 2013). Similarly, in differentiated cells such as osteoblasts and adipocytes, TNFα increases 11β-HSD1 activity to enhance local GC generation (Cooper *et al*. 2001, Tomlinson *et al*. 2010). Indeed, a range of cytokines and molecules, including IL1β and lipopolysaccharide (LPS), can stimulate 11β-HSD1 (Ishii-Yonemoto *et al*. 2010), suggesting that increasing 11β-HSD1 is a mechanism to initiate the process of inflammatory resolution through increased local GC generation. GCs are well documented to stimulate 11β-HSD1 expression and activity in most cell types including myocytes (Morgan *et al*. 2009). However, following muscle cell TNFα exposure, 11β-HSD1 activity is suppressed, with the effect mediated through p65 signalling and independent of secondary protein synthesis.

The best-established transcriptional regulators of *Hsd11b1* gene expression for a range of human and murine cell types are the members of CCAATT/enhancer-binding protein (C/EBP) family. *Hsd11b1* P1 promoter regulation of C/EBPα and C/EBPβ has been described previously (Balazs *et al*. 2008) and C/EBPα is regarded as a positive regulator of 11β-HSD1 transcription in hepatocytes with C/EBPβ acting as a repressor (Ignatova *et al*. 2009). However, in adipocyte and adipose tissue, C/EBPβ is an activator required to mediate the GC induction and cytokine regulation of 11β-HSD1 (Sai *et al*. 2008). More recently it has been established that an elevated ratio of C/EBPβ-liver enriched inhibitor protein and liver-enriched activator protein isoforms can downregulate 11β-HSD1 expression (Esteves *et al*. 2012). These data collectively represent an evidence for both the positive and negative regulation of 11β-HSD1 through transcriptional mechanisms in mature cells.

Here, we demonstrate that in both a murine skeletal muscle cell line and primary cultured myotubes 11β-HSD1 expression and activity are suppressed following TNFα exposure, with anisomycin experiments attributing this effect at the level of transcriptional regulation. This led us to search for potential TNFα-stimulated-NF-κB-binding motifs in the *Hsd11b1* P1 and P2 promoters, using the *cis*-regulatory element database (Robertson *et al*. 2006). Taking the three strongest targets from the *in silico* analysis, we used ChIP to identify a p65-binding sequence in the sequence motif located in the P2 promoter region (Fig. 5B) and associated this with the down-regulation of 11β-HSD1.

In contrast to our findings in muscle, adipocytes from p65 overexpression transgenic mice had elevated 11β-HSD1 at the mRNA and protein level, but show no evidence for direct gene regulation (Lee *et al*. 2013). In this context, NF-κB was acting as a positive regulator in a model of chronic systemwide p65 activation, indicating that 11β-HSD1 regulation is complex, with a number of tissue- and context-specific factors requiring consideration.

With this in mind, these data presented here represent the effects mediated following a single acute dose of TNFα, so biological interpretation in the context of that seen in another tissue type subjected to chronic TNFα stimulation should be cautious. TNFα-mediated regulation of 11β-HSD1 is part of a cellular response within muscle that targets a diverse set of transcriptionally regulated genes (Li *et al*. 2013). It may be that the suppressive effect observed is coordinated with the general suppression of myogenic differentiation, and dependent on interacting transcription factor and co-regulator availability. We have previously shown that in myoblasts response to TNFα by 11β-HSD1 is positively regulated, but in mature myotubes 11β-HSD1 is negatively regulated. This shift in regulation could in part be controlled at the level of *Hsd11b1* P1 and P2 promoters utilisation during commitment and progression through myogenic differentiation that ultimately determines overall 11β-HSD1 expression and activity.

We demonstrate that 11β-HSD1 suppression is observed in differentiated C2C12 and primary myotubes and emphasise the important role of coordinated *Hsd11b1* P1 and P2 promoters usage to control TNFα-regulated 11β-HSD1 activity. Further experiments are now required to expand this novel TNFα/NF-κB-mediated transcriptional suppression of 11β-HSD1 activity in the context of inflammatory disorders that can severely impact upon muscle structure and function.

## Author contribution statement

C L D, J B and A E Z conducted the work. P M S and G G L conceived and designed the research. C L D, M S C, P M S and G G L wrote the manuscript.

## Figures and Tables

**Table 1:** Primers used for ChIP detection of p65 binding

****	****
Putative response element 1 LEFT	ACCTGGGATGAACTGGATTG
Putative response element 1 RIGHT	ACTTTCTGTAGGCCTGTGTGC
Putative response element 2 LEFT	TCTGAGGCAAAGCCAAGACT
Putative response element 2 RIGHT	TAGCCAATCCAGCCATAACC
Putative response element 3 LEFT	GGTGAGCTCCCTTGCACTT
Putative response element 3 RIGHT	AGTTGCAACCCAGCCAGAC
NF-κB IκBα promoter LEFT	TAGCCAGCGTTTCCACTCTT
NF-κB IκBα promoter RIGHT	GGTCATGCACAGGGAACTTT
−10 kb 11β-HSD flanking LEFT	ACTAGCAATGTTCCCGCTGT
−10 kb 11β-HSD flanking RIGHT	AATGAGGGAATCTGGGGTTC
